# Interactive design generation and optimization from generative adversarial networks in spatial computing

**DOI:** 10.1038/s41598-024-54783-6

**Published:** 2024-03-02

**Authors:** Xiaochen Hu, Cun Lin, Tianyi Chen, Weibo Chen

**Affiliations:** School of Design and Innovation, China Academy of Art, Hangzhou, 310000 Zhejiang China

**Keywords:** Spatial calculation, Generative adversarial networks, Interactive design generation, Auxiliary classifier, Materials science, Mathematics and computing

## Abstract

This paper focuses on exploring the application possibilities and optimization problems of Generative Adversarial Networks (GANs) in spatial computing to improve design efficiency and creativity and achieve a more intelligent design process. A method for icon generation is proposed, and a basic architecture for icon generation is constructed. A system with generation and optimization capabilities is constructed to meet various requirements in spatial design by introducing the concept of interactive design and the characteristics of requirement conditions. Next, the generated icons can effectively maintain diversity and innovation while meeting the conditional features by integrating multi-feature recognition modules into the discriminator and optimizing the structure of conditional features. The experiment uses publicly available icon datasets, including LLD-Icon and Icons-50. The icon shape generated by the model proposed here is more prominent, and the color of colored icons can be more finely controlled. The Inception Score (IS) values under different models are compared, and it is found that the IS value of the proposed model is 7.05, which is higher than that of other GAN models. The multi-feature icon generation model based on Auxiliary Classifier GANs performs well in presenting multiple feature representations of icons. After introducing multi-feature recognition modules into the network model, the peak error of the recognition network is only 2.000 in the initial stage, while the initial error of the ordinary GAN without multi-feature recognition modules is as high as 5.000. It indicates that the improved model effectively helps the discriminative network recognize the core information of icon images more quickly. The research results provide a reference basis for achieving more efficient and innovative interactive space design.

## Introduction

In modern design, exploring methods for intelligent design processes has become increasingly important to improve design efficiency and creativity. Designers hope to utilize computer-aided technology to achieve more efficient and innovative design processes through automation and intelligence. Generative Adversarial Networks (GANs), as a powerful Deep Learning (DL) technology, have shown astonishing innovation potential in multiple fields^1,2^. Lv and Qiao^3^ pointed out that cognitive computing did not mean that computers replaced human thinking but rather made it an effective auxiliary tool for people to recognize and process large-scale data. How to fully utilize GANs to achieve interactive design generation and optimization to meet the constantly evolving design needs has become a hot research topic. As a creative field, spatial design requires not only meeting functional needs but also considering emotions, aesthetics, and user experience. However, traditional design processes are limited by manual operations and experience accumulation, making it difficult to efficiently implement complex design tasks. The emergence of GANs provides new possibilities for solving this problem. Through the game process of generators and discriminators, GAN can generate data samples with high fidelity, bringing revolutionary changes in design^4–6^.

In the past, the creativity and innovation of design mainly relied on the experience and intuition of designers, but this traditional approach often limited the diversity and efficiency of design. With the rise of data-driven methods, the role of computers in design has gradually become prominent^7,8^. GAN is an adversarial framework composed of generators and discriminators, which can generate realistic data by allowing two neural networks to play games with each other. This technology has achieved remarkable success in fields, such as image generation and speech synthesis, and its introduction into the design field has enormous potential. Spatial computing, as an important branch of the design field, covers multiple aspects, such as architecture, interior design, and urban planning. How to apply GANs in spatial design to achieve more innovative and diverse design generation and optimization has become a fascinating topic^9–11^.

In the application of various generative models, images, as a common data form with high information density, exhibit a wide range of possibilities. The transformation from landscape painting to oil painting, horses to zebras, and youth to elders and even the variability of facial expressions, the repair of damaged images, the improvement of image resolution, and the innovation of text-generated images have all enriched the application field of image generation^12^. These practical applications provide a multi-dimensional learning perspective, and image generation has become a crucial direction in generative models. With the advancement of artificial intelligence technology, the concept of automatically generating icons has gradually become a reality. Users can obtain preliminary ideas for various icons by inputting the required icon element features into the icon generation model. Then, users can select satisfactory icon styles and request designers to further refine them to ultimately create a satisfactory icon design.

This paper proposes an icon generation method based on GANs to meet the needs of spatial design. In this basic architecture, the generator and discriminator play games with each other to achieve high-quality icon generation. The concept of interactive design and requirement condition features are introduced to better adapt to the particularity of spatial design, and a system with generation and optimization capabilities is constructed. This paper introduces multi-feature recognition modules in the discriminator to maintain the diversity and innovation of the generated icons while meeting the conditional features. It is expected to effectively improve the diversity and fidelity of icon generation by optimizing the structure of conditional features.

## Literature review

At present, traditional image generation models typically use methods, such as image combination, cropping, color adjustment, and angle change, to create. With the advancement of DL neural network technology, the current research on generating models has focused on using neural networks to fit the distribution of raw image data. Then, a brand new image is generated based on this distribution. With the rapid development of DL technology, GAN has made significant research progress in interactive design generation and optimization in spatial computing. Gan et al.^13^ proposed an interactive urban layout generation method based on GAN. They used generators to generate candidate city layouts, and users could interact with the generation process by adjusting sketches in real-time. The discriminator evaluated the similarity between the generated layout and the real city layout, guiding the generation process and achieving collaborative design generation between users and the system. Niu et al.^14^ explored the method of using GAN to automatically generate product designs. The research team designed a generator network that used sketches as input to generate various possible product designs. The discriminator evaluated based on the existing product database, guiding the generator to generate more innovative and practical designs.

Hu et al.^15^ introduced a Three-Dimensional (3D) spatial data augmentation method based on auxiliary GAN. Researchers proposed SpatialGAN, which generated diverse 3D spatial data through generators to enhance the training dataset and improve the performance of DL-based spatial computing models. Dan et al.^16^ explored how to use deep generation models for interactive design space exploration. Researchers developed an interactive interface that allowed users to explore the design space while generating diverse design suggestions based on GAN to help users better understand and define design goals. Tang et al.^17^ conducted a comparative review on the application of different deep generation models in urban design. It covered the research progress of different models, such as variational autoencoders, GANs, and generative flows. Researchers evaluated the effectiveness and limitations of these methods in achieving interactive design generation and optimization from multiple perspectives.

Although existing methods have achieved interaction between user and generator, there are challenges in integrating user feedback. How to effectively integrate user feedback into the design generation and optimization process still requires further exploration. This paper focuses on how to more effectively integrate user feedback into the design generation and optimization process. The generator can more accurately understand the user's intentions and continuously optimize the generated design based on user feedback by developing an intelligent feedback mechanism.

## Materials and methods

### Icon generation model based on GANs

**Figure 1:**
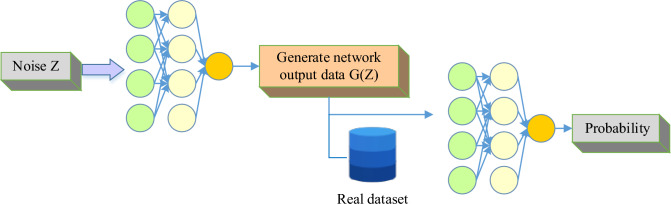
Structural schematic diagram of GAN.

The GAN is a network structure composed of generators and discriminators. The generator aims to generate samples similar to real data, while the discriminator aims to distinguish between the generated samples and real data. Through continuous adversarial learning, the generator gradually improves the fidelity of the generated samples, enabling them to be confused with the real. The core idea of GAN is to train the generated model through adversarial training of generators and discriminators^18–20^. The structural diagram of GAN is shown in Fig. 1. The left side of Fig. 1 shows the generated network G, with an input of a set of random noise Z and an output of new data G(z), aiming to make its distribution as close as possible to the real data distribution. The architecture of the generated network can be seen as the reverse structure of discriminant networks, creating new data with or without targeting through multi-layer deconvolution calculations. The successful quality of generating a network depends on the way it optimizes its structure and parameters. Training and sampling data are conducted in a generative network to measure the skill level of researchers in analyzing and calculating high-dimensional probability distributions.

The generator is a key component of icon GAN (IconGAN), whose task is to accept a random noise vector (usually represented as Z) and transform it into a new icon as similar as possible to the real icon. The generator adopts a multi-layer deconvolution network structure, gradually increasing the details and features of the icon to achieve icon generation. The optimization goal of the generator is to make the generated icons realistic enough to mislead the discriminator.

**Figure 2:**
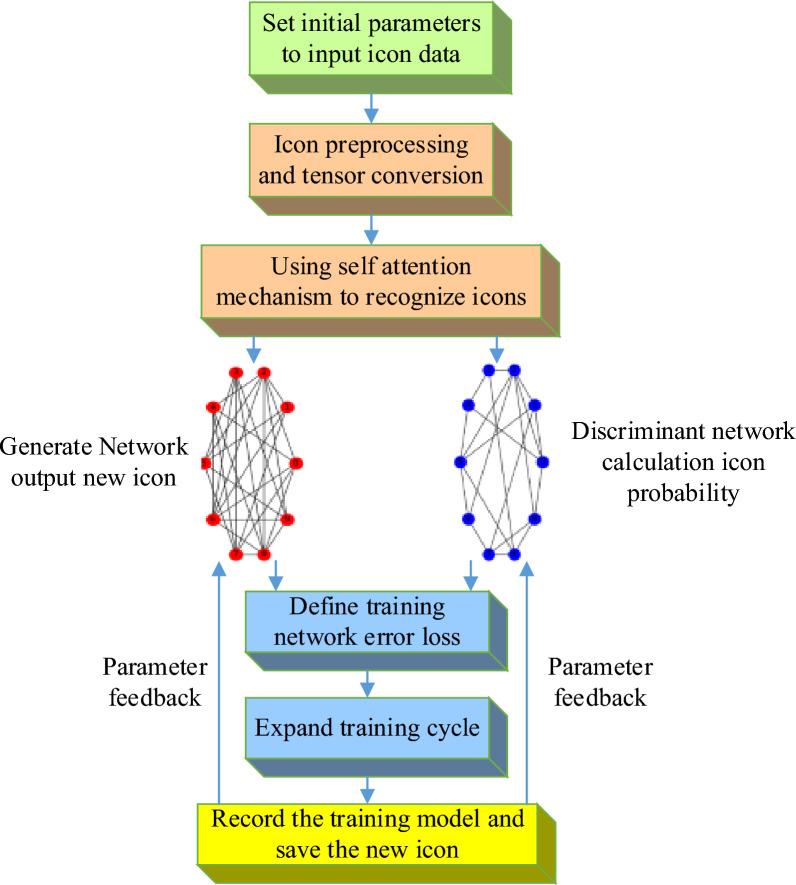
Basic framework for icon GANs.

The discriminator is another important component of IconGAN, whose task is to evaluate the similarity between the generated icon and the real icon. The input of the discriminator can be a real icon or an icon generated by the generator, with the goal of distinguishing between the two. The discriminator gradually learns how to distinguish between generated icons and real icons by judging between generated icons and real icons^21,22^. The optimization goal of discriminators is to improve their judgment ability to better distinguish the authenticity of generated icons. The training process of IconGAN is based on adversarial learning between the generator and discriminator. In each training iteration, the generator generates a set of icons, and the discriminator judges between these generated and actual icons. The goal of the generator is to deceive the discriminator by generating icons, making it difficult to distinguish between generated and real icons. The discriminator aims to accurately determine which icons are generated and which are real. Through continuous adversarial learning, the generator gradually improves the quality of generated icons, making them more realistic. The basic framework of IconGAN enables its widespread application in icon design^23–25^. IconGAN can generate high-quality icons similar to real icons by optimizing the structure and parameters of the generator and discriminator. The generated icons can be customized according to specific design requirements to meet the needs of different application scenarios, such as mobile applications, website design, and brand identification. Figure 2 shows the basic framework of icon GANs.

### Identification module incorporating multiple features

In traditional GAN, discriminators usually only rely on global features to determine the authenticity of the generated samples. This can easily lead to the generated samples that are not realistic enough in some details, and unstable phenomena, such as pattern collapse and pattern collapse, can also occur during the training process^26,27^. Multi-scale discriminator is an improved approach that can capture image information more comprehensively by introducing discriminators of different scales. In the image generation task, discriminators can be trained on the original image and the image with reduced size simultaneously^28–30^. This multi-scale discriminator can capture features at different levels from details to the global, improving the authenticity and diversity of generated samples.

The multi-level feature discriminator proposed here is a structure that designs the discriminator as multiple sub-discriminators. Each sub-discriminator is responsible for different levels of features, such as low-level textures and high-level semantics. This design enables the discriminator to analyze images at multiple levels of abstraction, thereby improving the judgment ability and diversity of generated samples. A multimodal discriminator is a method that integrates multiple features and considers different aspects of an image, such as appearance and semantic information. A multimodal discriminator is a model component designed to handle multiple data modalities or features. In DL, this may involve multiple neural network branches, each specialized in processing a specific data type (such as images, text, and sound). The outputs of these branches may be integrated into a common representation or used for different tasks, such as classification and generation. The multimodal discriminator helps the model understand and utilize the relationships among different data types, thereby improving the model's performance. The model can simultaneously focus on multiple aspects of the image by introducing a multimodal discriminator, thereby more accurately evaluating the authenticity of the generated samples. This helps generate more creative and diverse samples.

**Figure 3:**
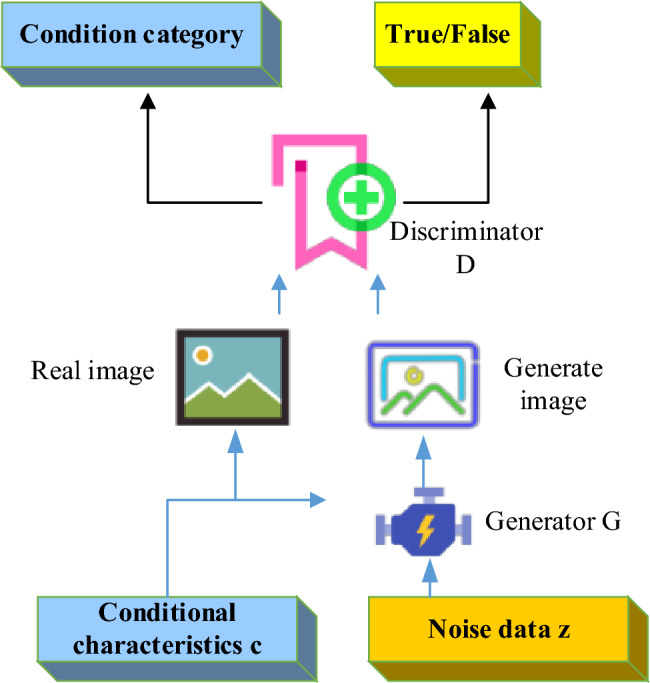
Icon generation model based on ACGAN.

The improved Auxiliary Classifier GAN (ACGAN) has significant differences from the auxiliary GAN. In ACGAN, the generator still receives conditional features, but the design of the discriminator differs from traditional methods. In the discriminator, conditional features are no longer directly input but are added to the output through the conditional feature recognition module. This makes the task of the discriminator clearer, with the goal of checking whether the input image meets the conditional features expected by the generator. Therefore, in ACGAN, the generator is responsible for generating images that meet the conditional features, while the discriminator is responsible for verifying whether the images meet the conditions. The model structure is given in Fig. 3. This method more effectively captures important conditional features of the image, such as color and shape, making the generated image closer to reality.

With the Wasserstein GAN (WGAN) model, the stability and generation efficiency of ACGAN can be further improved^31,32^. The distance among distributions is measured using Wasserstein distance. It introduces color condition features and the shape condition feature *s*. In this way, the Auxiliary Classifier Wasserstein GAN (ACWGAN) model is formed, and the loss functions of the generator and discriminator are:

1 $$L_{D} = E_{{z\sim p_{z} \left( z \right)}} \left[ {f\left( {G\left| {z\left( {c,s} \right)\left| {c,s} \right.} \right.} \right) - E_{{x\sim p_{{r\left| {c,s} \right.}} \left( {x\left| {c,s} \right.} \right)}} f\left( {x\left| {c,s} \right.} \right)} \right]$$

2 $$L_{G} = - E_{{z\sim p_{z} \left( z \right)}} \left[ {f\left( {G\left| {z\left( {c,s} \right)\left| {c,s} \right.} \right.} \right)} \right]$$

The function $f$ follows the constraint of Lipschitz constant, and the parameters of this function are obtained through neural network training. To meet the limit of Lipschitz constant, the parameter $\omega$ is constrained to a pre-set cutoff parameter $h$ during each round of parameter updates. In addition, the conditional features of the model are composed of color condition feature $c$ and shape condition feature $s$, which can collectively affect the generation of icons. If necessary, further features can be added to make the generated icons more targeted.

After selecting and constructing the icon generation model, it is necessary to clarify the training method and evaluation strategy of the model. Here, the initial ACGAN is improved, and a multi-class GAN model is proposed. The algorithm process is as follows. Batch training is adopted for iterative training, which involves extracting data from the dataset to form batches for training. The specific training process is shown in Table 1.

**Table 1:** Training process based on ACWGAN model.

**Input:** learning rate α, cutoff parameter $h$, batch parameter *m*, rounds of identification of one generation *n*_*c*r*i*t*ic*_, *z*_*size*_ noise length, *w*_0_ initial discriminator parameter, $\theta$ initial generator parameters, shape condition features $s$, color condition feature $c$
1. Initialization parameters
2. **while**
3. **for** t = 0, …,* n*_*c*r*i*t*ic*_ **do** // discriminator
4. Collect a batch of data from a real dataset $\left\{ {x^{\left( i \right)} } \right\}_{i = 1}^{m} \sim P_{r}$
5. Generate a batch of data according to distribution $\left\{ {z^{\left( i \right)} } \right\}_{i = 1}^{m} \sim P_{r}$
6. Collect a batch of data from the color and shape condition label y, which are $\left\{ {c^{\left( i \right)} } \right\}_{i = 1}^{m}$,$\left\{ {s^{\left( i \right)} } \right\}_{i = 1}^{m}$
7. **update** $$\omega \leftarrow \omega + \alpha RMSProp\left( {\omega ,g_{\omega } } \right)$$
8. Control parameters within the specified range $\omega \leftarrow clip\left( {w, - h,h} \right)$
9. **end for**
10. Generate a batch of data according to distribution $\left\{ {z^{\left( i \right)} } \right\}_{i = 1}^{m} \sim P\left( z \right)$ // generator
11. **update** $$g_{\theta }$$
12. **update** $$\theta$$
13.** end while**

### Construction of an icon generation network for conditionally assisted improvement

Conditional inputs, usually specified features or parameters, are introduced into the icon generation network to customize the generated icons. These conditions can be color, shape, size, and style. Conditional input guides the generator to generate icons with specific styles or features, achieving customized generation. The appropriate generator and discriminator network architecture is selected to adapt to conditional inputs. The generator needs to combine conditional input with random noise to generate icons that match the conditions. The discriminator needs to determine whether the generated icon meets the conditions^33,34^.

In the generation network, the previously obtained label needs to be converted into a label tensor label (label_tensor) suitable for matrix calculation, which is simultaneously passed in a flat layer and an embedding layer to convert the two-dimensional output matrix into a one-dimensional vector. Subsequently, the number of labels (num_classes) is combined. Then, it is combined with noise through the superposition function to generate input. Finally, the input is merged with the label tensor (label_tensor) to form a new input for the generated network. Therefore, the conditional variable C is regarded as the limiting design concept in icon design, which brings constraints to the design direction and output results of the training network.

Relatively speaking, the processing of discriminative networks is simpler. When processing inputs and conditions, operations are carried out through a fully connected layer to ensure that the output dimension is consistent with the number of labels. Next, after a single-layer normalization operation, the results are transmitted to the training process. In the formal training phase, the labels are first randomly sampled to obtain the sampled labels (sampled_labels) and generate new icons (gen_imgs). d_loss_real calculates the loss between the input imgs and the original label (img_labels), using the valid values (valids) as the input. d_loss_new evaluates the loss between the newly generated icon (gen_imgs) and the sampled label (sampled_labels), and the input is a false value (fake).

## Experimental design

During the model training process, a series of key parameter settings are involved, which play a crucial role in the performance and effectiveness of the model. The optimization function settings of the WGAN model are referenced, and RMSprop is used as the optimization function. The learning rate of the neural network is set to 0.00005, while the batch size of the data is set to 32. Besides, the parameter update process of the neural network refers to the cutoff parameter of the WGAN model, set to 0.01. In the specific construction of the model, the activation function of the generator adopts Rectified Linear Unit (ReLU), while the output of the generator follows the Tanh activation function in the WGAN model. To better train the discriminator, Leaky ReLU is used as the activation function in its convolutional layer, and the parameter value is set to 0.2.

The specific network parameter settings are shown in Table 2, which aim to achieve better convergence and generation effect during the training process. The selection of these parameters has undergone sufficient experiments and optimization to ensure that the improved model can achieve satisfactory performance in path planning problems.

**Table 2:** Parameter settings based on ACWGAN.

Network layer		Convolutional kernel	Step	Depth	Dropout	Activation function	Normalization
Input:G(z)-100	Dense	–	–	128*7*7	0	–	ReLU
	Reshape	–	–	7,7,128	0	–	–
	UpSampling	–	–	–	0	–	–
	Convolution	4 × 4	1 × 1	128	0	Yes	ReLU
	UpSampling	–	–	–	0	–	–
	Convolution	4 × 4	1 × 1	64	–	Yes	ReLU
	Convolution	4 × 4	1 × 1	3	–	–	Tanh
D(x,z)-28*28*1	Convolution	3 × 3	2 × 2	16	0.25	Yes	-Leaky ReLU
	Convolution	3 × 3	2 × 2	32	0.25	Yes	Leaky ReLU
	Convolution	3 × 3	2 × 2	64	0.25	Yes	Leaky ReLU
	Convolution	3 × 3	1 × 1	128	0.25	Yes	Leaky ReLU
	Flatten	–	–	–	–	–	–
	Dense	–	–	1	–	–	–

The Python-based machine learning framework Tensorflow is adopted to build the model, and the testing platform for building the model is configured accordingly. The configuration of the testing platform includes a 3.9 GHz Intel Core i7 CPU, Nvidia RTX-2070 8 GB GPU, and 32 GB of memory.

Two different icon datasets are used in the experiment, namely Icons-50 and LLD-Icon. The LLD-Icon dataset is a large-scale icon dataset created by researchers, such as Alexander Sage. Unlike traditional icon datasets, the characteristic of the LLD-Icon dataset is that it is collected from the internet and contains many real-world icon samples. This dataset contains 486,377 icons, each with an image size of 32 × 32 pixels with 3 color channels. This means that the image of each icon is an RGB color image. The Icons-50 dataset is another commonly used icon dataset, mainly used for icon recognition and image classification tasks. The Icons-50 dataset is manually annotated, and each icon is assigned to a predefined 50 categories. Each category represents a different type of icon, and each icon image is also a 32 × 32 pixel RGB color image.

After obtaining the dataset, it must be preprocessed to adapt to the input criteria of the model. Firstly, the size of image data is standardized to 28 × 28 pixels. The data is divided into two groups: color and black and white to conduct a more comprehensive experiment. In the black and white icon group, the Opencv image processing library is used to convert color images into black and white images. The Scikit-learn scientific computing library is used to divide the dataset into training and testing sets, with the testing set accounting for 20% of the total sample size.

## Results and discussion

### Analysis of icon generation effect

**Figure 4:**
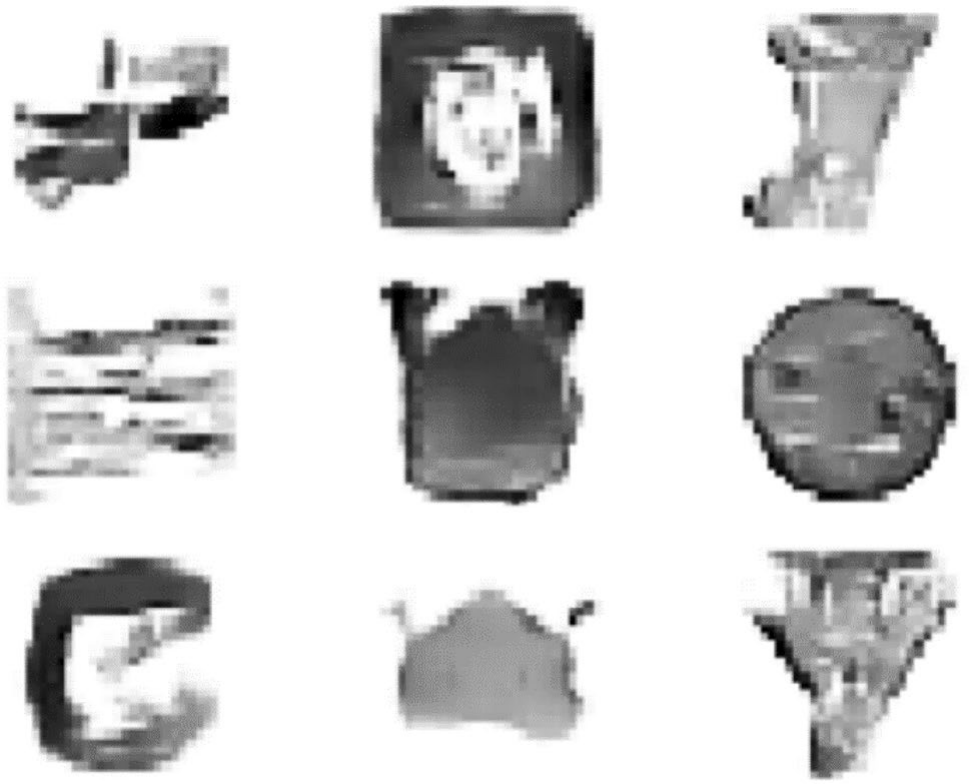
LLD-Icon black and white icon generation rendering based on ACWGAN.

**Figure 5:**
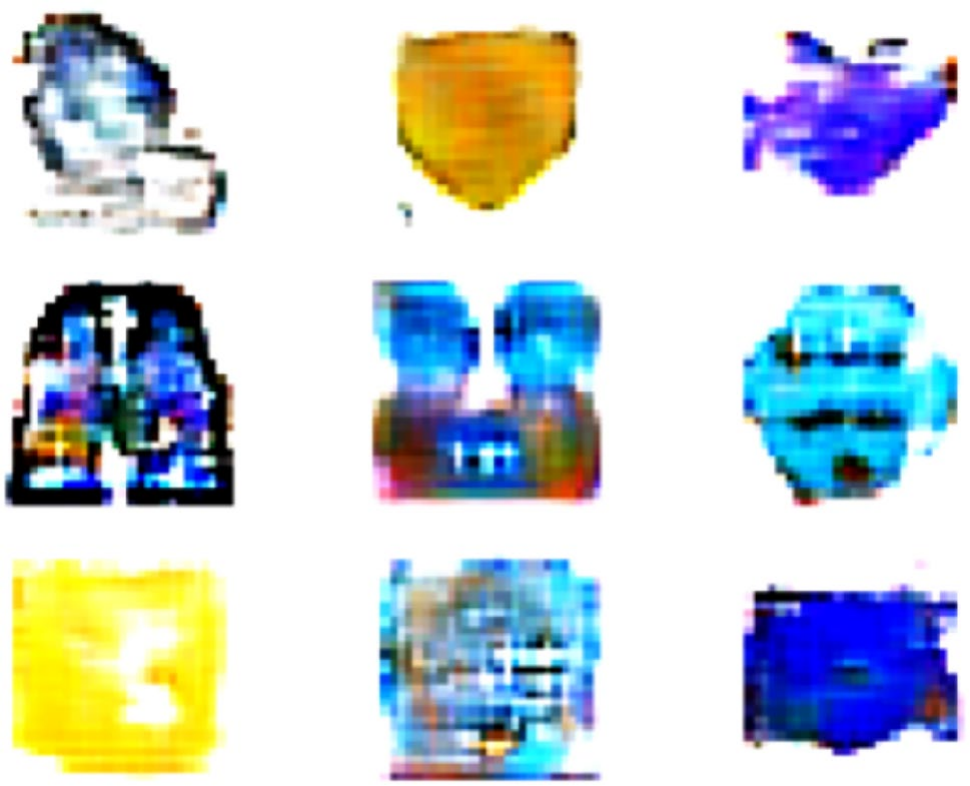
LLD-Icon color icon generation rendering based on ACWGAN.

**Figure 6:**
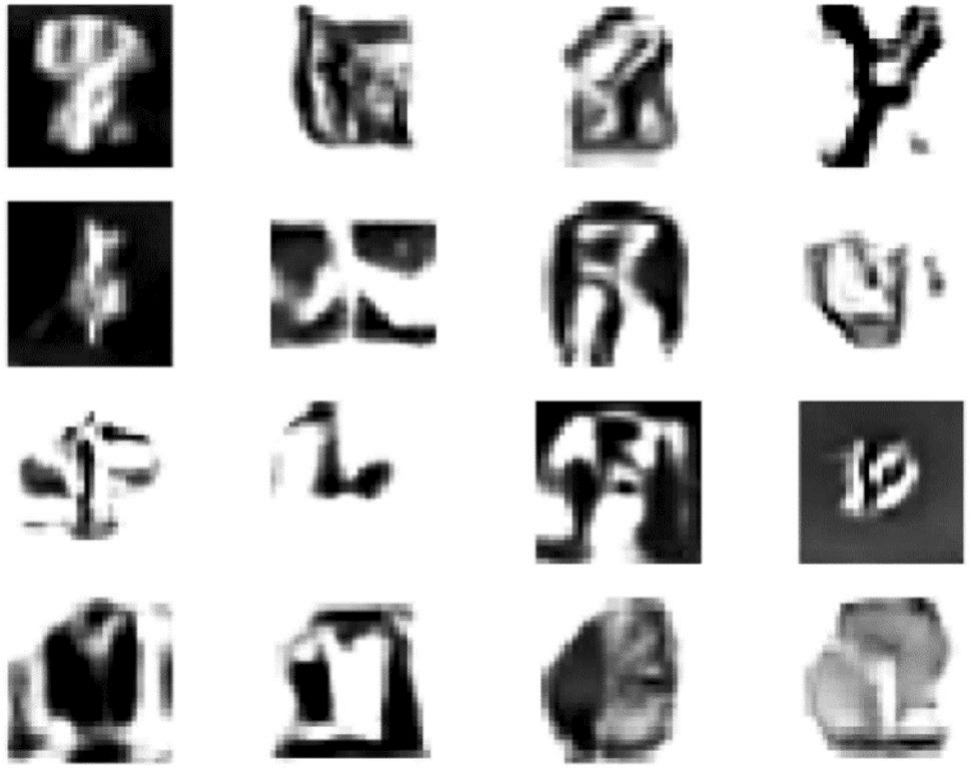
Icons-50 black and white icon generation rendering based on ACWGAN.

**Figure 7:**
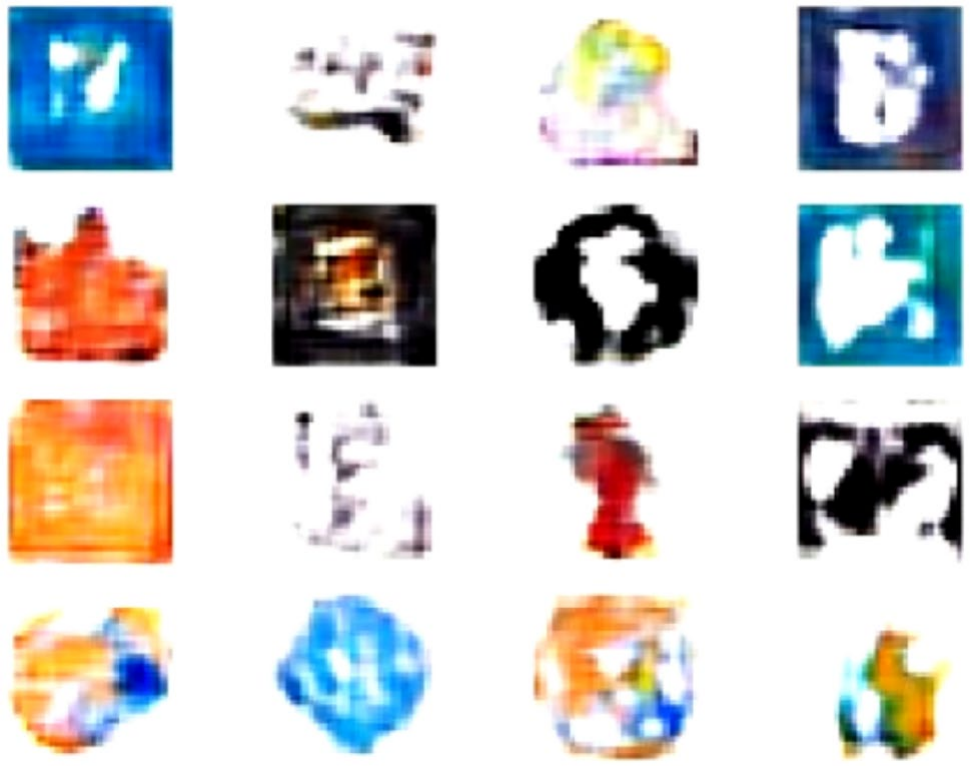
Icons-50 color icon generation rendering based on ACWGAN.

The ACWGAN proposed here is validated on the Icons-50 and LLD-Icon datasets for the generation of black and white and color icons. The specific results are revealed in Figs. 4, 5, 6 and 7. The experimental results show that the conditional GAN model with conditional constraints has stronger directionality based on GAN models. Especially in generating black and white icons, the model can more prominently highlight shape features. The generated bicycle icon can be clearly seen, which fully demonstrates the important role of conditional features in icon generation. Compared to using only GAN models, the improved model incorporating conditional features is more capable of generating icon styles with more target features based on specific conditions. This also indirectly verifies the effectiveness of the conditional GAN model in icon generation tasks.

### Comparison of Inception Score (IS) and Fréchet Inception Distance (FID) values for different generation models

**Figure 8:**
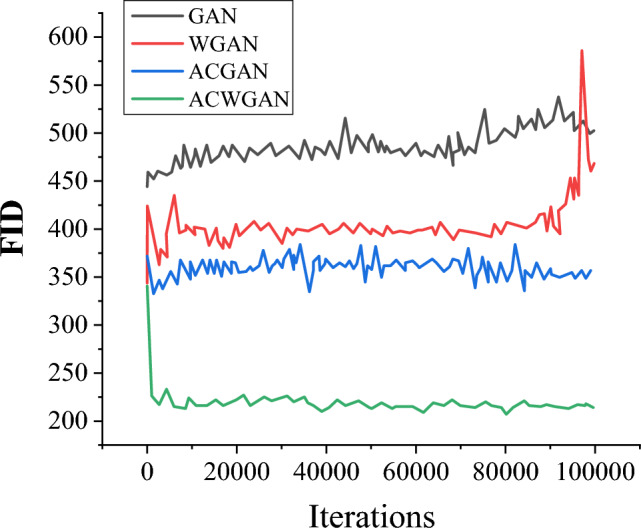
Comparison of FID values for different generation models.

**Figure 9:**
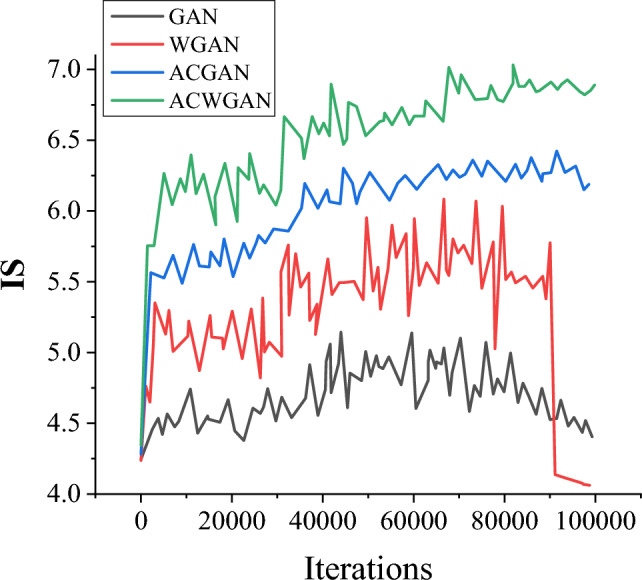
Comparison of IS values for different generation models.

Figures 8 and 9 show the dynamic changes in FID and IS values of the GAN model, WGAN model, ACGAN model, and the proposed ACGAN model on the Icons-50 dataset. Generally speaking, a smaller FID value indicates that the distribution of generated samples is closer to the distribution of real samples. Additionally, the higher the IS value, the higher the authenticity score of the generated samples, and the higher the clarity of the generated samples. These two evaluation indicators jointly reveal the superiority of the generated model, and the generated samples can better simulate the distribution of real data and have higher performance in terms of quality and clarity.

After a series of iterations, the FID value of the ACWGAN model rapidly decreases, while the IS value gradually increases to 7.05, resulting in the generated image gradually becoming clearer. As the iteration continues, the mutual confrontation between the generative model and the discriminative model leads to a continuous improvement in the quality of the generated image. At around 100,000 iterations, the ACWGAN model tends to stabilize, while other comparative models are slightly unstable and even experience model crashes. This leads to a sharp increase in FID values and a sharp decrease in IS values. Moreover, after introducing multi-feature recognition modules into the model, the peak error of the recognition network is only 2.000 in the initial stage, while the initial error of the ordinary GAN without multi-feature recognition modules is as high as 5.000. It indicates that the improved model effectively helps the recognition network recognize the core information of the icon image faster.

## Conclusion

This paper explores the application potential and optimization problems of GANs in spatial computing and proposes an interactive design generation and optimization method based on multiple features. It constructs a basic architecture by establishing icons, introduces the concept of interactive design and requirement condition features, and constructs a system with generation and optimization capabilities, aiming to meet the diverse needs of different spatial design fields. The generated icons have successfully achieved greater diversity and innovation while maintaining conditional features by integrating multi-feature recognition modules into the discriminator. The experimental results show that the multi-feature icon generation model ACWGAN proposed here performs well in shape highlighting and color fine control and demonstrates its potential application in spatial design.

Although this paper introduces conditional features and multi-feature recognition modules to optimize the generation process, there is still some optimization space in practical applications. For example, how to better determine and design conditional features and how to select and fuse multiple features are issues that require further research. In addition, more GAN structures can be explored, such as variational autoencoder GANs, to further improve the performance of the generation model.

### Supplementary Information

Supplementary Information.

## Data Availability

All data generated or analyzed during this study are included in this published article [and its supplementary information files].
