# Supplementary figures and images for: Interactive design generation and optimization from generative adversarial networks in spatial computing
Source: Sci Rep. 2024 Mar 2;14:5154. doi: 10.1038/s41598-024-54783-6 (PMC10908823; doi:10.1038/s41598-024-54783-6)

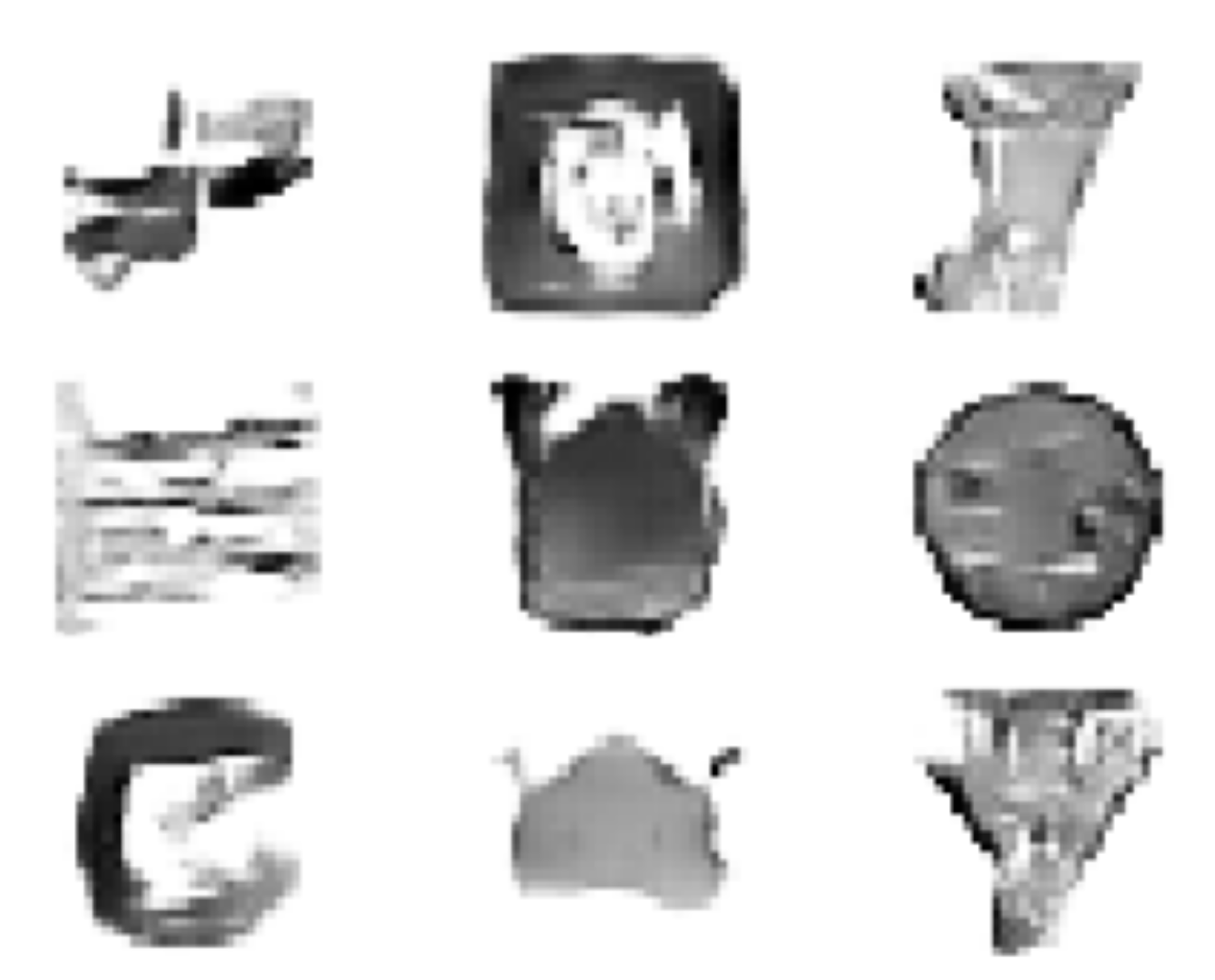

Supplement: Supplementary file 1 — Supplementary Information. [file 41598_2024_54783_MOESM1_ESM.zip › Data packet/Figures/figure4.png]

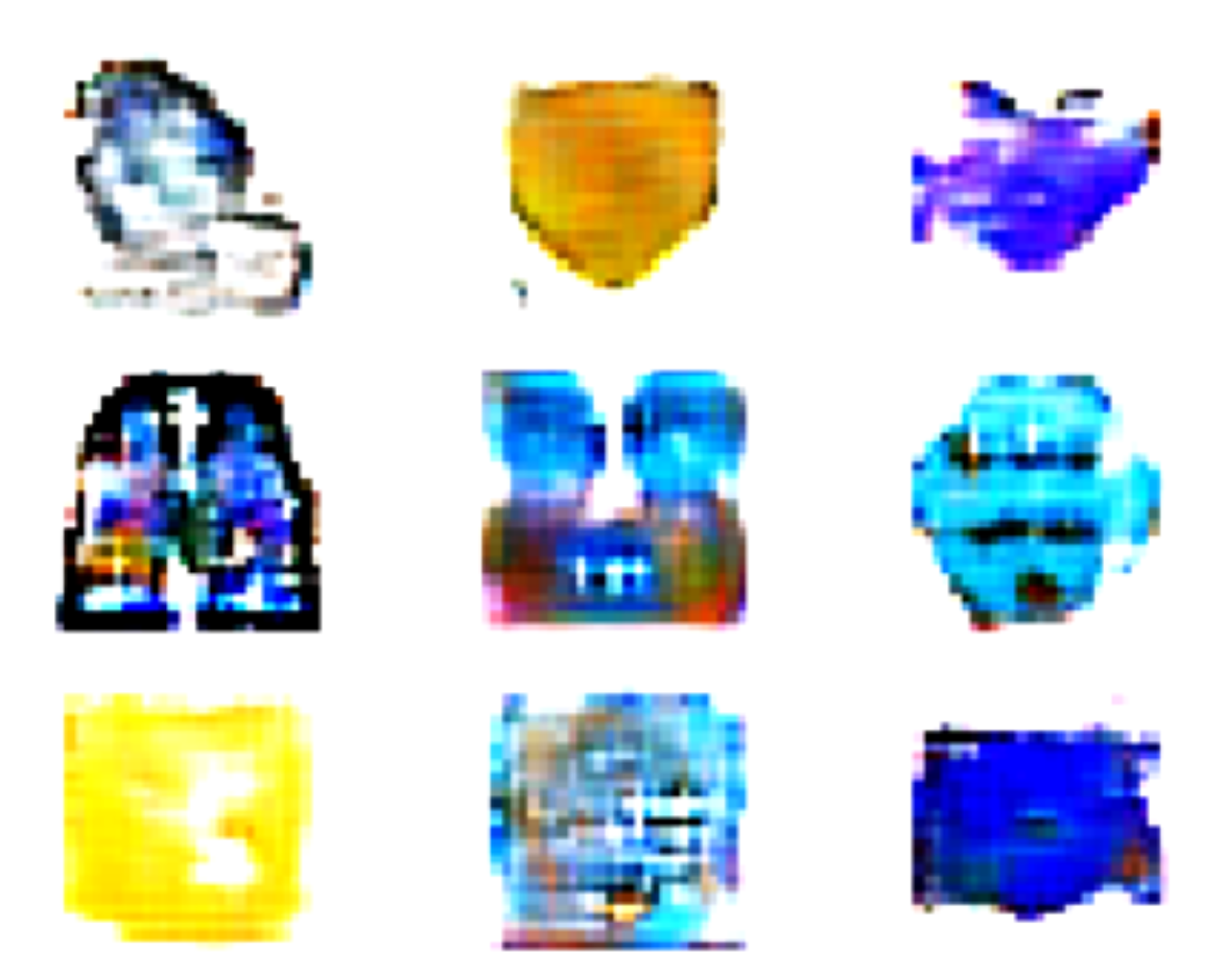

Supplement: Supplementary file 1 — Supplementary Information. [file 41598_2024_54783_MOESM1_ESM.zip › Data packet/Figures/figure5.png]

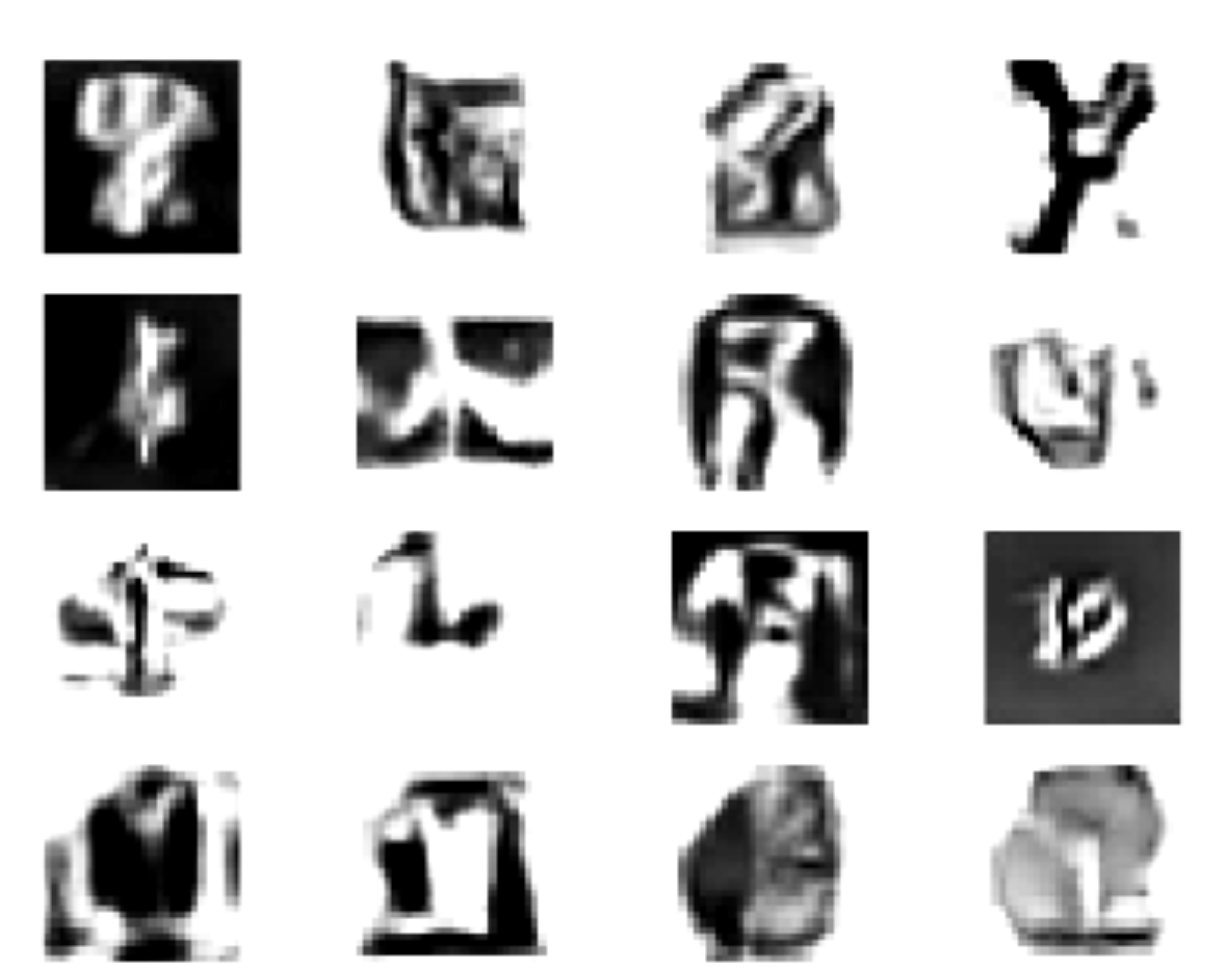

Supplement: Supplementary file 1 — Supplementary Information. [file 41598_2024_54783_MOESM1_ESM.zip › Data packet/Figures/figure6.png]

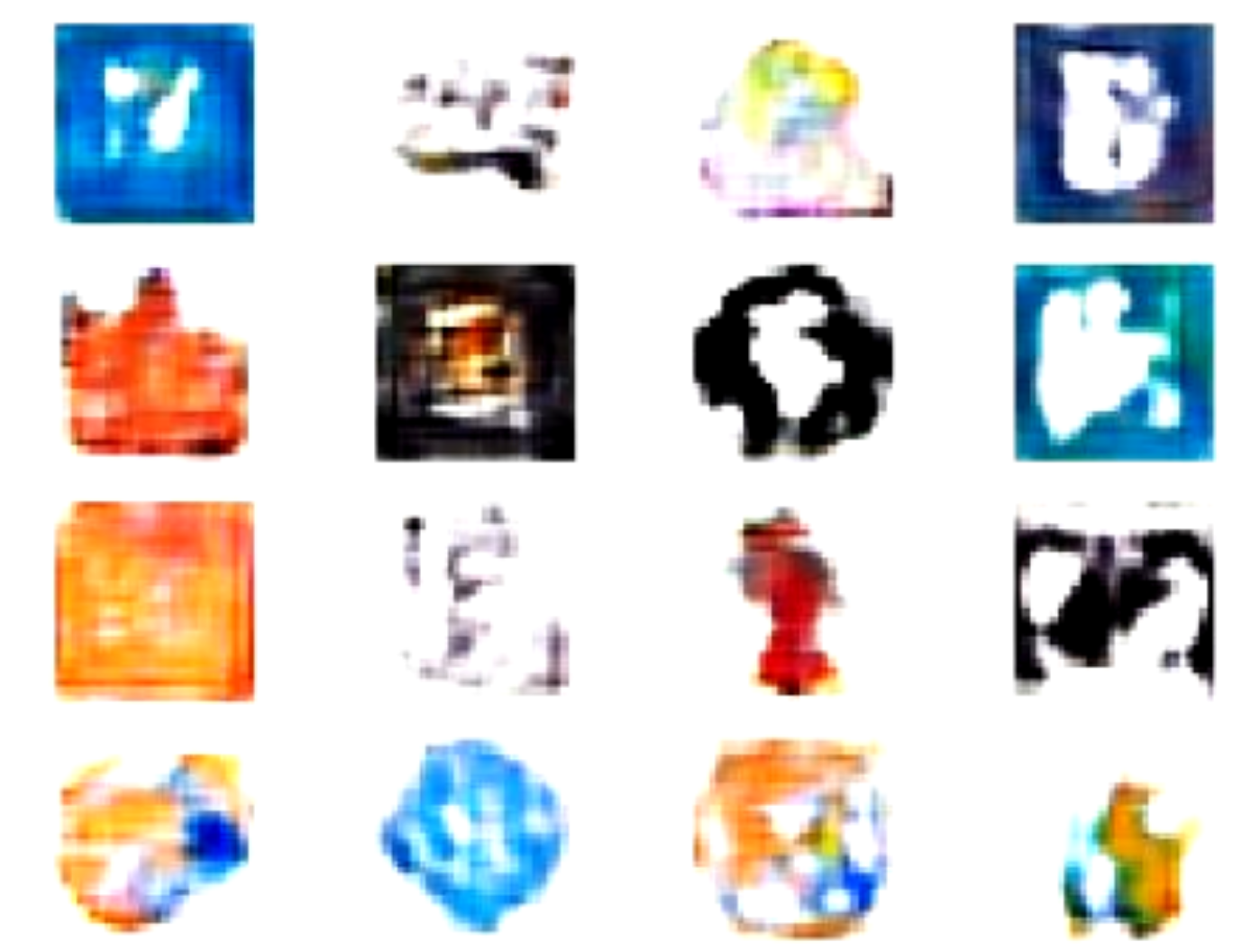

Supplement: Supplementary file 1 — Supplementary Information. [file 41598_2024_54783_MOESM1_ESM.zip › Data packet/Figures/figure7.png]
